# Identification of Small-Molecule Inhibitors of *Yersinia pestis* Type III Secretion System YscN ATPase

**DOI:** 10.1371/journal.pone.0019716

**Published:** 2011-05-18

**Authors:** Wieslaw Swietnicki, Daniel Carmany, Michael Retford, Mark Guelta, Russell Dorsey, Joel Bozue, Michael S. Lee, Mark A. Olson

**Affiliations:** 1 The Uniformed Services University, Bethesda, Maryland, United States of America; 2 Battelle Memorial Institute, Battelle Eastern Science and Technology Center, Aberdeen, Maryland, United States of America; 3 Research, Development and Engineering Command, Edgewood Chemical and Biological Center, Aberdeen Proving Ground, Maryland, United States of America; 4 Division of Bacteriology, United States Army Medical Research Institute of Infectious Diseases, Fort Detrick, Maryland, United States of America; 5 Department of Cell Biology and Biochemistry, United States Army Medical Research Institute of Infectious Diseases, Fort Detrick, Maryland, United States of America; Queen Mary University of London, United Kingdom

## Abstract

*Yersinia pestis* is a Gram negative zoonotic pathogen responsible for causing bubonic and pneumonic plague in humans. The pathogen uses a type III secretion system (T3SS) to deliver virulence factors directly from bacterium into host mammalian cells. The system contains a single ATPase, YscN, necessary for delivery of virulence factors. In this work, we show that deletion of the catalytic domain of the *yscN* gene in *Y. pestis* CO92 attenuated the strain over three million-fold in the Swiss-Webster mouse model of bubonic plague. The result validates the YscN protein as a therapeutic target for plague. The catalytic domain of the YscN protein was made using recombinant methods and its ATPase activity was characterized *in vitro*. To identify candidate therapeutics, we tested computationally selected small molecules for inhibition of YscN ATPase activity. The best inhibitors had measured IC_50_ values below 20 µM in an *in vitro* ATPase assay and were also found to inhibit the homologous BsaS protein from *Burkholderia mallei* animal-like T3SS at similar concentrations. Moreover, the compounds fully inhibited YopE secretion by attenuated *Y. pestis* in a bacterial cell culture and mammalian cells at µM concentrations. The data demonstrate the feasibility of targeting and inhibiting a critical protein transport ATPase of a bacterial virulence system. It is likely the same strategy could be applied to many other common human pathogens using type III secretion system, including enteropathogenic *E. coli*, *Shigella flexneri*, *Salmonella typhimurium*, and *Burkholderia mallei*/*pseudomallei* species.

## Introduction

The Gram negative bacterium, *Yersinia pestis*, is a zoonotic pathogen responsible for epidemics of human plague [1]. The pathogen can be spread by flea bites, causing bubonic plague, or through aerosol exposure, causing pneumonic plague. There is no approved vaccine licensed for human use in the United States and to be effective, antibiotics have to be given within 24-hr post-exposure to the aerosolized form of pathogen. In view of the potential for natural and artificial resistance to the current antibiotics, alternative forms of treatments, preferably compatible with the existing therapeutics, are of interest to the medical community. Recent interests of researchers are focused on many aspects of the infection process, including blocking transcriptional activation, inhibiting function of proteins secreted by bacteria during infection, or disabling the full virulence system by interference with its assembly and function. In many Gram negative bacteria, including *Y. pestis*, the type III secretion system (T3SS) is the preferred target for novel therapeutics development using the described strategies [2], [3], [4], [5], [6], [7], [8], [9]. The system has been studied extensively and there is a direct evidence for its essential role in virulence in *Y. pestis* and other pathogens [10], [11]. The system is encoded on a plasmid, pCD1 in *Y. pestis*, and is conserved across species. Structurally, the organization is very similar to the bacterial flagellar system and many proteins are direct homologues of the flagellar system [12]. *Yersinia pestis* assembles the outside shell, the injectisome, composed of *Ysc* proteins (*Yersinia* secretion chaperone) to deliver *Yop*s (*Yersinia* outer proteins) directly from the bacteria into the mammalian cell (review in [13]). The delivery is cell-contact-dependent and one of the primary host targets are macrophages. The secreted proteins, which include YopH, a phosphatase, and YopE, a potent activator of human Rho GTPases, disrupt the cellular signaling network enabling *Yersinia* to survive intracellular and to potentially be spread through macrophages [14], [15]. The mechanism of Yops delivery is known in general but the fine details are not clear. In the bacterial cytoplasm, many Yop effectors (YopE, YopH, YopB, YopD, YopO/YpkA, and YopT) are made in complex with *Syc* (specific *Yersinia* chaperone) proteins to prevent degradation and keep them in a partially unfolded state. The partial unfolding, confirmed by structural data, is presumed to be necessary for transport through the pore as the measured pore diameter is not sufficient to allow for transport of fully folded proteins [16], [17]. The removal of chaperones is facilitated by a single ATPase and requires ATP hydrolysis [18]. In the plant-like T3SSs, the homologous HrcN ATPase forms a double hexameric head-to-head assembly located in the center of the entrance to the translocation pore [19]. In the animal-like T3SSs, which include *Y. pestis* system, the ATPase is most likely attached to the side of the translocation pore [20]. It is hypothesized that the oligomeric, most likely hexameric, form of the ATPase in the animal-like T3SS is necessary for its biological activity [21]. The energy source for the transport of the proteins through the pore is not known. In the flagellar system, a proton gradient has been proposed as the potential energy source [22], but this hypothesis is still controversial.

The structural and functional conservation of the T3SSs across many pathogens has made it an attractive target for novel antibacterial therapeutics development with broad spectrum activity. In the enteropathogenic *Escherichia coli*, EscN, a homolog of YscN, is essential for virulence in the animal mode of infection, and deletion of the gene or a mutation in the catalytic site of the protein abolishes the delivery process of virulence factors [23]. In *Y. enterocolitica*, deletion of the *yscN* gene abolishes secretion of all Yop effectors in a bacterial cell culture model [24]. Deletions in the animal-like T3SS in *Burkholderia mallei*, a zoonotic pathogen responsible for glanders and a CDC select agent [25], [26], decreased the virulence of the pathogen at least two orders of magnitude in an animal model [25]. Because *B. mallei* also has a type VI secretion system (T6SS) essential for virulence [27], the data may reflect partial attenuation.

Current strategies for T3SS inhibition strategies do not specifically target the T3SS ATPases [2], [3], [4], [5], [6], [7], [8], [9] due to concerns of a future therapeutic cross-reacting with human enzymes. However, the bacterial enzymes have less than 25% identity to human ATPases and the active sites show significant differences between bacterial and human enzymes. In this work, effort was focused on the YscN ATPase as the target for interference with the function of the T3SS in *Y. pestis*. The *yscN* gene was shown to be essential for virulence of *Y. pestis* in a mouse model of bubonic plague as deletion of the region coding for the catalytic domain of the YscN ATPase totally attenuated the pathogen. Therefore, the catalytic domain of the recombinant enzyme was purified under native conditions as a fusion with a maltose-binding protein (MBP) and characterized biochemically. The protein had ATPase activity which required Mg^+2^ for its activity. To help design potential small-molecule inhibitors of the enzyme, a database of commercially available drug-like molecules was computationally screened against the active site. The best candidates from a small test set were able to fully inhibit the YscN ATPase activity in an *in vitro* assay at micromolar concentrations. The same compounds also inhibit the homologous *B. mallei* BsaS ATPase activity in an *in vitro* assay at similar concentrations. In addition, the small molecules prevent secretion of the YopE effector by attenuated *Y. pestis* into the bacterial medium and mammalian cells at micromolar concentrations. The current work shows the feasibility of targeting T3SS ATPases towards the future development of novel, broad-spectrum bacterial therapeutics.

## Results

### The yscN gene is essential for plague virulence

**Figure 1:**
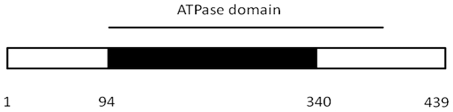
*yscN* gene deletion in *Y.pestis* CO92. The *yscN* gene fragment (black box) was deleted in the pCD1 plasmid as described under Materials and Methods. The approximate position of ATPase domain is marked with the bar on the top of the box. Numbers below the box correspond to approximate positions in the amino acid sequence marking the boundaries of the deleted region.

**Figure 2:**
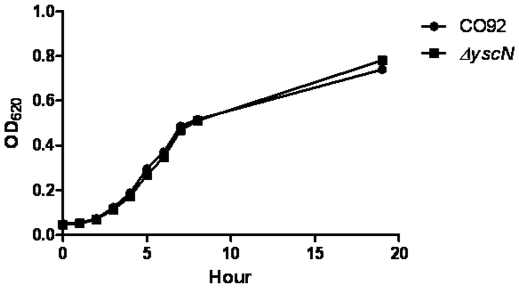
Deletion of the yscN gene in *Y. pestis* CO92 strain does not affect the growth of bacteria. Fresh dilutions of the wild type and *ΔyscN* strains of bacteria were grown at 28°C as described under Materials and Methods. A graph for one of the representative experiments is presented.

The high functional conservation of T3SS in bacteria and the presence of a single ATPase per virulence system suggest a critical role for the ATPase protein in the function of T3SS. In other bacteria, deletion of the orthologous ATPase abolishes the secretion of virulence factors thereby decreasing the virulence of the pathogen [10], [28]. The deletion of the *Y. pestis* CO92 YscN ATPase would be expected to at least decrease the secretion of virulence factors, including Yops and LcrV, and possibly attenuate the virulence of *Y. pestis* CO92. Indeed, the in-frame deletion of the *yscN* gene (Fig. 1) caused total attenuation of the pathogen. The LD_50_ for the s.c. route of delivery was greater than 3.2×10^6^ CFU in the Swiss-Webster model of plague (Table 1). The deletion did not affect the growth rate of the mutant *in vitro* (Fig. 2) which confirms that the deletion does not affect overall metabolism of the bacteria under *in vitro* conditions. The results confirm a very well known fact that the TTSS is essential for virulence but not for survival of bacteria outside the host [29]. The LD_50_ for the s.c. route of delivery of the Δ*yscN Y. pestis* CO92 mutant was greater than 3.2×10^6^ CFU in the Swiss-Webster model of plague (Table 1), which is in a strong contrast to the well established value of LD_50_ of 1–2 CFU for the wild-type *Y. pestis* CO92 bubonic plague infection model in Swiss-Webster mice [30]. Attempts to calculate a complete LD_50_ were not successful as higher doses could not be used due to the physical limitation of the delivery method. None of the animals died within the 21 days after inoculation and no signs of discomfort (ruffled fur, excessive grooming, and signs of morbidity) were observed during the course of the experiment. Due to the well-established lethality of *Y. pestis* and to reduce unnecessary animal use, a single dose of 180 CFU of the wild-type pathogen was used as a positive control. None of the animals from the control group survived beyond the 21 days post-inoculation observation period. In contrast, all the animals inoculated with up to 3.2×10^6^ CFU survived for 21 days post-inoculation. These current data prove that the *yscN* gene is essential for *Y. pestis* virulence and the observed attenuation is not due to the interference with the overall metabolism of *Y. pestis*.

**Table 1:** The ΔyscN gene deletion in *Y. pestis* CO92 creates a fully attenuated ΔyscN CO92 strain in a mouse model of bubonic plague.

Dose	Survival after 21 days
*CFU*	*alive/total*
0	10/10
32	10/10
322	10/10
3220	10/10
32200	10/10
322000	10/10
3220000	10/10
32000000	10/10
Control	0/10*

Groups of 10 Swiss-Webster female mice were inoculated s.c. with the deletion mutant and observed for 21 days as described under Materials and Methods. Data are statistically valid (p<0.05). For control experiments, the animals were challenged with 180 CFU of wt *Y. pestis* CO92 strain as described under Materials and Methods.

### Fusion of YscN catalytic domain with maltose binding protein retains ATPase activity despite oligomerization

Expression and purification of the ATPase-active form of the YscN protein proved to be a challenging protein engineering effort. The YscN protein in *Y. pestis* is predicted to be an Mg^2+^-dependent ATPase. The full length protein expressed very poorly in *E. coli* and had to be fused to a permanent membrane anchor to increase yield. This version was not suitable for characterization as the detergent interfered with ATPase activity of the protein. Instead, an N-terminal fusion with MBP was chosen to avoid the presence of detergents. This strategy is commonly used for studying difficult-to-express proteins and MBP solubility tag has been used to express many functional ATPases [31], [32], [33]. The MBP fusion of wild-type YscN protein was stable in *E. coli* but tended to aggregate and the yield was very low. The yield was not improved when the *yscN* gene was optimized for *E. coli* expression and all of the three Cys residues were replaced by Ser to prevent aggregation due to non-native intermolecular disulfide bridge formation. However, shortening of the expression construct to the catalytic domain region amino acid residues 94–419, removal of the C-terminal 20 residues and a replacement of the single Cys residue with Ser improved the yield dramatically and retained the ATPase activity of the recombinant protein. The MBP affinity tag was not removed as cleavage with proteases necessitated inclusion of detergents to prevent aggregation. The optimized catalytic domain fragment, residues 94–419, fused to the MBP anchor, was used throughout all the experiments. The fragment will be referred to as ‘optimized YscN catalytic domain’ in the current work.

The optimized YscN catalytic domain purified as an oligomeric species. The major peak eluted at 118 mL on a HiLoad Superdex 200 26/60 gel filtration column, which corresponded to a hydrodynamic radius of a hexamer or a higher-order oligomer. The smaller peaks constituted less than 10% of the main peak area and did not contain the optimized YscN catalytic domain protein as examined by SDS-PAGE.

**Figure 3:**
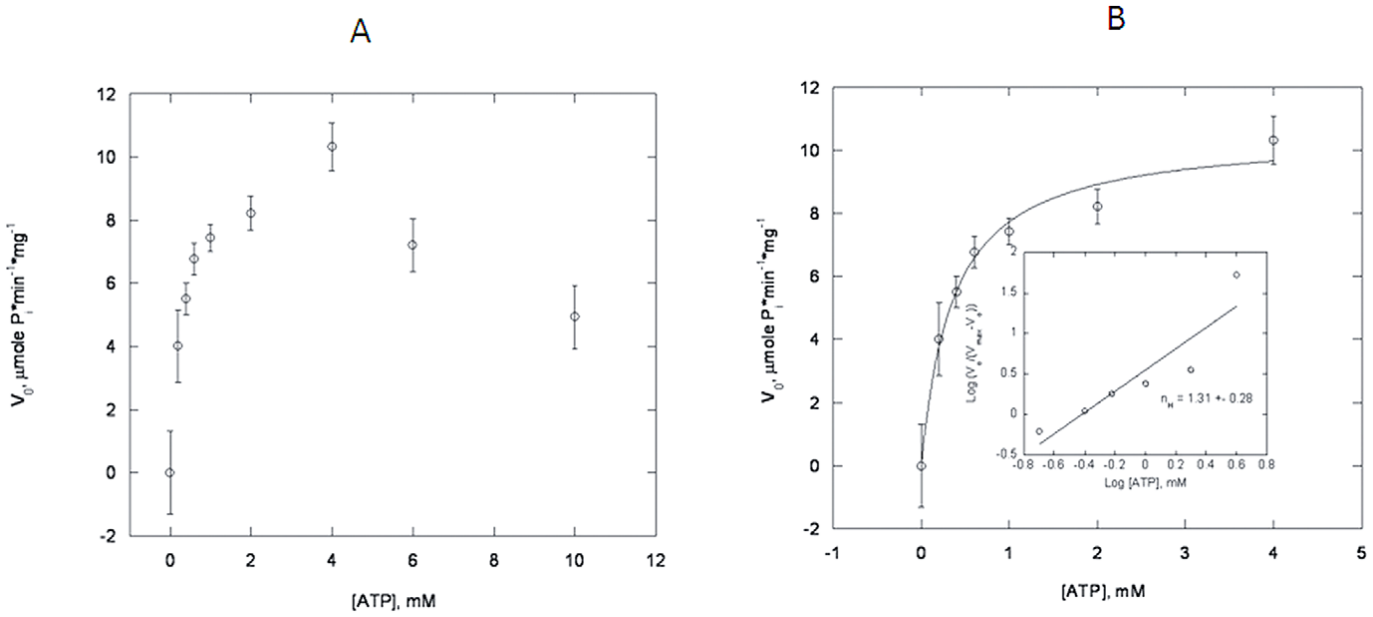
Steady-state kinetics of ATP hydrolysis by the optimized catalytic domain. (A) The ATP hydrolysis is inhibited above 4 mM ATP concentration. (B) The hydrolysis of ATP by the enzyme shows a positive cooperativity up to 4 mM ATP concentration. The kinetics of hydrolysis was measured by following phosphate release in 10 mM Tris, pH = 7.6, 150 mM NaCl, and 1 mM Mg^+2^ at 37°C as described under Materials and Methods. Total protein concentration was 9.6 µg. Error bars correspond to standard deviation of triplicate measurements.

The observed aggregation could be a result of a severe misfolding of the YscN protein or reflect a non-specific oligomerization due to the hydrophobic properties of the YscN protein. The severely misfolded protein would exhibit a minimal ATPase activity as compared to other reported homologous proteins. To investigate the issue in detail, we examined the kinetics of the ATP hydrolysis. The initial velocity of phosphate release from ATP increased with the initial concentration of substrate up to about 4 mM (Fig. 3A). After that, the initial rate decreased which is observed commonly in kinetics measurements at over-saturating enzyme concentrations. The kinetics up to [ATP] = 4 mM showed a positive cooperativity with a Hill's coefficient of 1.31±0.28 (Fig. 3B), similar to the data observed for the homologous EscN enzyme [34]. Steady-state analysis of the kinetics determined apparent *V*_max_ and *K*_m_ values of 10.52±0.51 µmole P_i_/min/mg of enzyme and 0.36±0.06 mM, respectively. Corrections for the cooperativity did not improve fit quality and were not included in the final apparent *K*_m_ and *V*_max_ calculations. The specific ATPase activity of the YscN catalytic domain (Fig. 3 A) was about an order of magnitude higher than reported for the homologous EscN protein [34] but about two orders of magnitude lower than reported for the *Y. enterocolitica* YscN enzyme [35]. The optimized catalytic domain of YscN has measured ATPase activity within the fairly broad reported range of specific activities of various other ATPases. In summary, the data preclude a gross misfolding of the protein and show positive cooperativity by the enzyme during ATP hydrolysis.

### Inhibition of YscN optimized catalytic domain by small molecules

**Figure 4:**
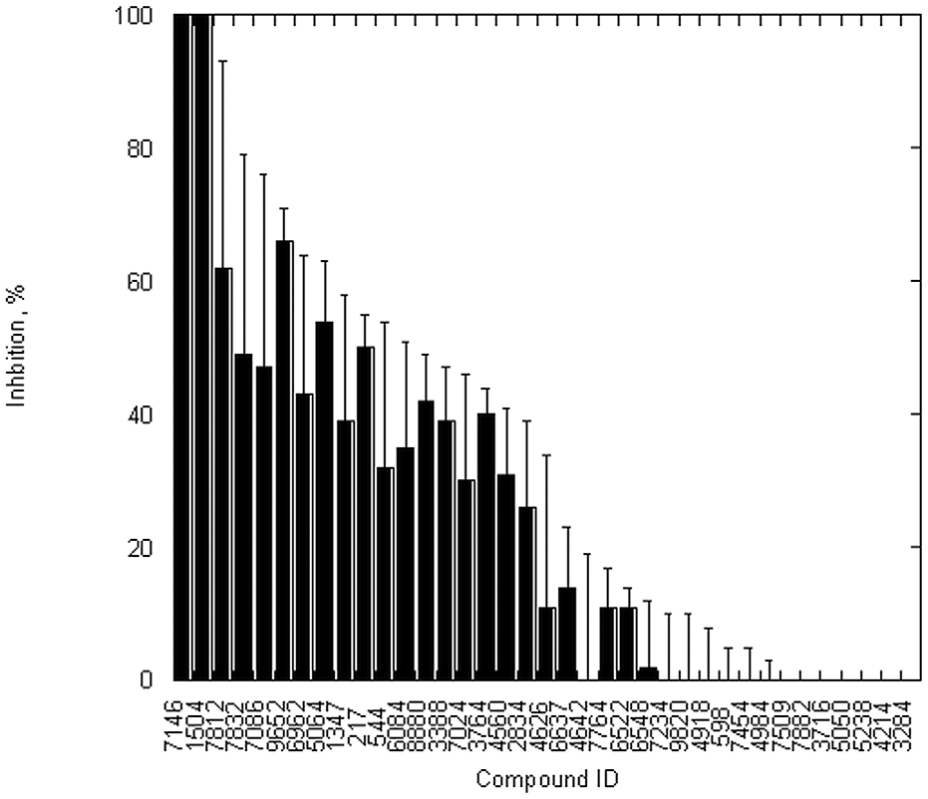
Small-molecule inhibitors are capable of fully blocking the optimized catalytic domain ATPase activity. The small molecules derived from computational screen of ZINC database were incubated with the enzyme at 37°C and ATP hydrolysis was determined by measuring ADP release as described under Materials and Methods. Inhibitor concentrations were 100 µM for all reactions. The numbers on the X axis correspond to in-house IDs for individual compounds. Error bars correspond to standard deviation of triplicate measurements.

To help us search for the inhibitors of the *Y. pestis* T3SS ATPase, we performed computational screening of a virtual 3D database [36] of drug-like molecules against a comparative protein model of the active site of YscN. The first phase of the search identified about 20,000 compounds, while the second phase led to the selection and purchase of 50 compounds with LigScore2 (Accelrys) values> = 6.8. The hydrophobic nature of the compounds prevented characterization of YscN inhibition at values greater than 100 µM ligand concentrations. Due to the solubility problems, only 37 of the purchased compounds (Appendix S1) were used in biological assays. Attempts to use alternative methods (high/low pH, high temperature) for the rest of the compounds were not successful as the compounds precipitated from the reaction mixtures during activity assays. The first screen of the enzyme using the soluble compounds identified several compounds capable of fully inhibiting the ATPase activity at 100 µM concentration (Fig. 4). A detailed analysis of the inhibition determined that several compounds could inhibit 50% of the ATPase activity below 40 µM concentration (Table 2). The best ones, compounds with ID numbers 7146 and 1504, were effective below 20 µM concentration. The data were not affected by the amount of DMSO used in the assay and the results were repeated many times in independent measurements, demonstrating stability of the compounds in the solvent and reliability of the assays. The only exception was compound with ID number 4640 which changed its inhibitory properties after prolonged storage in DMSO at room temperature. Compound 3624 was not used in the ATPase inhibition assays due to the interference of intrinsic fluorescence with the Adapta (Invitrogen) assay.

**Table 2:** IC_50_ values^a^ for inhibition of ATP hydrolysis by YscN optimized catalytic domain for selected small molecules.

Compound ID	IC50, µM
7812	41 b
7146	16.5±1.5
1504	18.2±4.9
7086	32 a
9652	50 a
7832	70 a

The ATPase inhibition was measured by following ADP release at 37°C as described under Materials and Methods.

a The value was determined from the best fit as the concentration at which 50% inhibition of ATPase activity was observed.

### Small molecules effective against YscN ATPase cross-inhibit homologous *B. mallei* BsaS ATPase

The *B. mallei* BsaS ATPase shares over 40% sequence identity with *Y. pestis* YscN ATPase and the active site is almost identical in both pathogens based on homology modeling. It has been shown for many pathogens using the same type of virulence factors delivery systems that compounds effective against one species are effective against other species [37]. Accordingly, we hypothesized that the best molecules effective against *Y. pestis* T3SS ATPase would have an effect on the homologous *B. mallei* T3SS near-full-length BsaS ATPase. Indeed, the hypothesis was confirmed experimentally (Table 3). The small differences in IC_50_ values likely reflected small changes in the active site of the proteins as both ATPases were purified under native conditions using the same strategy. The results show feasibility of developing broad-spectrum inhibitors targeting T3SS ATPases.

**Table 3:** Top inhibitors of YscN ATPase cross- inhibit ATP hydrolysis by near-full-length BsaS ATPase.^a^

Compound	IC50
In-house ID	µM
7812	23±15
7146	7.4±0.9
1504	28.4±7.4

a The ATPase inhibition was measured by following ADP release at 37°C as described under Materials and Methods. The values were determined as the concentrations at which 50% inhibition of ATPase activity was observed.

**Figure 5:**
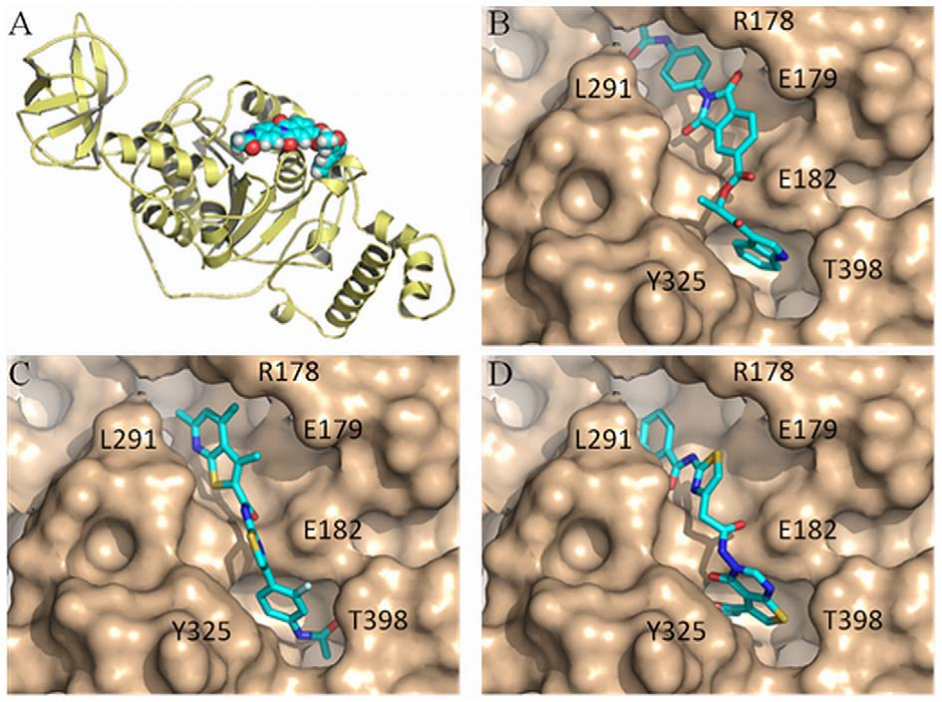
Structural model of YscN ATPase with compounds docked into the active site. (A) Protein fold of the YscN model (colored yellow) built by comparative modeling methods and illustrated with the docked compound ID number 7812 (depicted by atomic spheres). (B) Molecular surface of the YscN model showing the active-site region (colored gold) bound by compound ID 7812 in its conformational pose (stick representation, where in general the color cyan are carbon atoms, nitrogen atoms are blue, oxygen are red, and sulfur are yellow). (C) Modeled YscN complex with compound ID 7146. (D) Modeled YscN complex with compound ID 7086.

The comparative protein model of YscN used in the *in silico* screening effort and models of the best inhibitors docked onto the protein surface are illustrated in Fig. 5. The compounds shown are ID 7812, 7146, and 7086. The docked conformational poses are overall similar among the molecules, yet their chemical composition and the details of their interaction with YscN are different. The binding pocket flanked by YscN residues Y325 and T398 is occupied by ring structures of the small molecules and the binding interactions reflect potential π-π stacking with the tyrosine ring. This tyrosine-based pocket is the binding site for the adenine moiety of ADP as observed in various crystallographic structures of ATPases and ATP synthases, including the structure used to build our comparative model of YscN. The compound 7146 extended well into the YscN pocket by placing chemical groups that exhibit strong polarity, and thus may help to explain the more favorable IC_50_ for this molecule. The cavity at YscN residues R178 and E179 is the location of binding the phosphate groups of ADP. The compound 7812 extends into this cavity and provides surface complementarity through multiple ring structures.

### Small-molecule ATPase inhibitors are effective in blocking YopE secretion by *Y. pestis* in a bacterial cell culture

**Figure 6:**
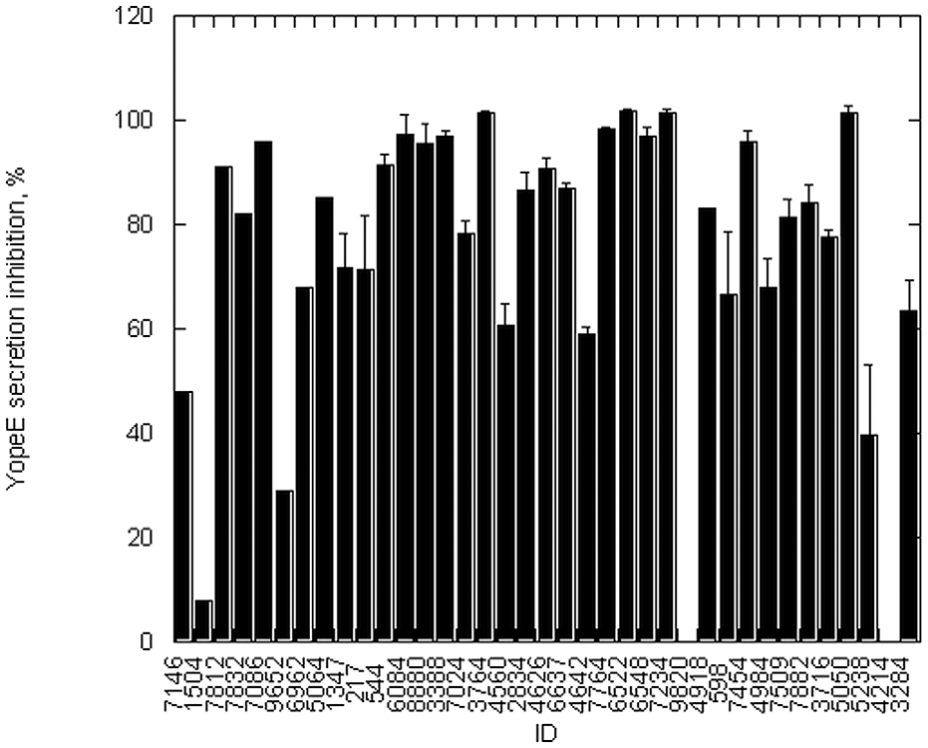
Inhibition of YopE secretion by selected small molecules. The secretion of YopE was measured in the supernatants of bacterial cell culture by ELISA using anti-YopE antibody as described under Materials and Methods. The data were collected 1 hr post-induction with EGTA. Inhibitor concentrations were 100 µM. Positive and negative controls correspond to the results obtained for co-solvent (DMSO) without and with EGTA, respectively. Error bars correspond to standard deviation of five measurements.

The small-molecule compounds shown to be effective against YscN and BsaS ATPase were tested in a cell culture assay for inhibition of the secretion of virulence factors. Many of the compounds inhibiting YscN ATPase activity (Fig. 4) also inhibited the secretion of YopE by the attenuated *Y. pestis* (Fig. 6). The compounds with ID numbers 7146 and 1504 had the lowest IC_50_ (Table 2) but were poor inhibitors of the YopE secretion (Fig. 6). The compounds with ID numbers 7812, 7086, and 7832 were also good inhibitors of YopE secretion (at least 50% inhibition at 50 µM or lower concentration, Fig. 6) and had the IC_50_ estimates between 30 and 70 µM (Table 2).However, the compound 9652 was poor in the secretion inhibition assay (30% at 100 µM concentration, Fig. 6). The three compounds: 7812, 7832 and 7086, showed a good correlation between inhibition of YopE secretion and the ATPase activity and were selected for further studies. There were several compounds which showed excellent YopE secretion inhibition at 100 µM concentration despite marginal performance in the ATPase inhibition assay: compound IDs 4642, 6548, 7234, 4918, 598, 7454, 4984, 7509, 7882, 3716, 5050, 5238, and 3284. The compound 3624 showed excellent performance in the YopE secretion assay but was unsuitable for the ATPase inhibition assays. The compounds with IDs 4214 and 9820 showed lack of inhibition in both assays. The differences may be due to many factors discussed below.

The overall correlation between the activity and secretion assays was very poor (Student t-test, paired data: p<0.006). The reason for the discrepancy between the two assays is not clear but may involve ability of the compounds to enter bacterial cells and interact with the YscN ATPase, metabolism inside the bacteria, efflux from the pathogen, and interaction with other bacterial proteins, including those important for T3SS function and assembly. The last option may be an explanation for the compound 4918, among others.

The difference cannot be explained by misfolding of the protein as both BsaS and YscN ATPases, with only 40% identity, were inhibited by the same compounds with a similar potency (Table 3). Possible explanations may include binding outside the catalytic pocket of YscN and affecting assembly of biologically important complexes or targeting sterically similar sites in other bacterial proteins important for regulating the effector secretion [38], [39], [40], [41].

To help us find a correlation between the chemical activity and blockage of bacterial secretion, we performed chemical similarity analysis on the 29 compounds that had YopE secretion inhibition, EC_50_, better than 100 µM. The largest cluster of active compounds all contained a thiophene group. A smaller cluster contained molecules in which a sulfonyl group bridges an aniline and phenyl ring. The most noticeable feature amongst the top hits for EC_50_ and enzymatic activity (IC_50_) was the presence of a 6-member ring fused to a 5-member ring, which is topologically similar to the purine/adenine moiety found in ADP and ATP. The best inhibitors may then represent chemical mimics of the bacterial ATPase substrate.

**Figure 7:**
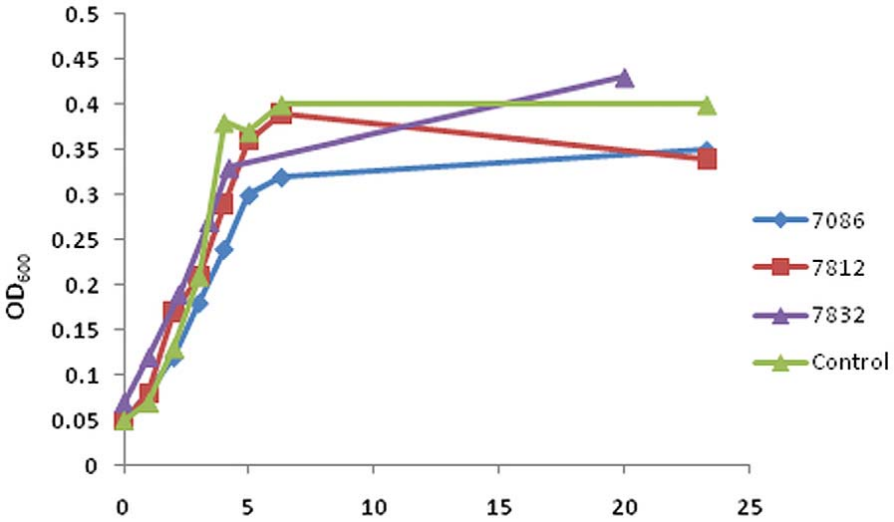
Inhibition of *Y. pestis* KIM growth by selected small molecules. Attenuated *Y. pestis* KIM19 strain was grown in the presence of 100 mM inhibitor in HBI medium at 30°C as described under Materials and Methods. Control experiment was performed with 2% DMSO. Data were normalized to the starting OD_620_ = 0.15 at 0 hr time. For a reference, the secretion of YopE protein (Fig. 6) was measured at t = 3 hrs.

The differences shown between *in vitro* and *in vivo* activities (Fig. 4 and Fig. 6) could also be caused by the inhibitors' influence on the general bacterial growth. We selected compounds 7812, 7832 and 7086 as the most promising compounds based on their combined scores from YopE secretion and ATPase inhibition assays and further analysis discussed below. Bacterial growth in the presence of selected inhibitors 7832 and 7812, showed that none of them had a significant effect on the growth rate of *Y. pestis* in a bacterial medium (Fig. 7) except for the compound 7086 which had a small bacteriostatic effect. The results confirm that the selected small molecules, except maybe for the compound 7086, do not affect the general metabolism of *Y. pestis* and exclude slower growth rate of bacteria in the presence of inhibitors as the cause of YopE secretion inhibition.

The bioavailability of the compounds was not tested directly in the current work. Indirectly, we performed a second screen of YopE secretion inhibition at 10 µM inhibitor concentration for the best 21 compounds tested at 100 µM (Fig. 6). Only three compounds: 7812, 7832, and 7086, would inhibit YopE secretion at 95% or higher levels (Table 4). The compounds were only moderate (45–60%) inhibitors in the ATPase activity assay (Fig. 4). The compounds 7812 and 7832 also showed minimal bacterial growth inhibition at 100 µM concentration (Fig. 7) but the compound 7086 had some bacteriostatic effect. The observed inhibition of YopE secretion is most likely related to the inhibition of T3SS ATPase activity (compounds 7812, and 7832) except for the compound 7086. Compound 7086 inhibits the ATPase activity but it also has a slight bacteriostatic effect. In this case, the inhibition of secretion may be due to some interference with the bacterial growth.

**Table 4:** Secondary screen of YopE secretion inhibition by selected small molecules.

Inhibitor	Inhibition at 10 µM
*ID*	*%*
DMSO+EGTA	0±8
7086	106±0.5
7832	97±0.4
7812	103±0.2

The secretion of YopE was measured in the supernatants of bacterial cell culture by ELISA using anti-YopE antibody as described under Materials and Methods. The data were collected 1 hr post-induction with EGTA. Inhibitor concentrations were 10 µM. Positive and negative controls correspond to the results obtained for co-solvent (DMSO) without and with EGTA, respectively. Error bars correspond to standard deviation of five measurements. Errors correspond to standard deviation of five measurements.

The next step in compound screening was determination of cytotoxicity on mammalian cells alone by the top overall scoring compounds (Table 4). At 100 µM concentration, the compounds 7812, 7832 and 7086 showed minimal general cell toxicity against HeLa cells (Table 5) while inhibiting at least 80% the YopE secretion in a bacterial cell culture (Fig. 6), making them potential therapeutic candidates. However, compound 7086 exhibited a noticeable toxic effect and would have to be modified as a potential therapeutic candidate. Compound 3624 showed about 20% cell toxicity at 100 µM concentration.

**Table 5:** Cytoxicity of selected small molecules against human HeLa cells.

Inhibitor	Inhibition at 100 µM
*ID*	*%*
Methanol	100±1
DMSO	0±20
7086	26±11
7832	0±8
7812	0±18

The top overall scoring inhibitors were incubated at 100 µM concentration with HeLa cells as described under Materials and Methods. The toxicity was measured by the ability of cells to metabolize calcein-AM using LIVE/DEAD kit (Invitrogen). Methanol and 2% DMSO were used as positive and negative controls, respectively. Error bars correspond to S.E.M from triplicate measurements.

The last step in the screening process was determination of the ability to block the toxic effects to mammalian cells by the molecules. Delivery of effectors by *Y. pestis* causes morphological changes in the mammalian cells due to the actin depolimerization mainly by YopE [42]. The infected cells change their normal morphology and become rounded up due to the destruction of mammalian cytoskeleton. We took advantage of this fact to quantitate the ability of *Y. pestis* to secrete effectors in the presence of small molecules. The top overall scoring compounds were not able to fully prevent the change in cell morphology at 50 µM concentrations (Table 6). However, additional screens of compounds showing activity in YopE secretion screen into bacterial cell culture identified one compound, 3624, which was able fully inhibit cytotoxicity effects to mammalian cells at 10 and 50 µM concentration. The compound was not identified in the ATPase screen as its intrinsic fluorescence prevented use of the Adapta assay. A small group of the compounds, including the compound 4214, was only marginally effective in the cell infection model.

**Table 6:** Inhibition of HeLa cell toxicity by attenuated *Y. pestis* in the presence of selected small molecule inhibitors.

Inhibitor	Inhibition at 50 µM	Inhibition at 10 µM
*ID*	*%*	*%*
7086	20	0
7832	30	0
7812	0	0

The HeLa cells were incubated with attenuated *Y. pestis* KIM19 strain in the presence of small molecules in DMEM medium supplemented with 10% FBS as described under Materials and Methods. Cell toxicity was scored by calculating the percentage of rounded versus the squamous cells under a microscope. Only data for molecules showing 10% or more inhibition of cell rounding at 50 µM inhibitor concentration are shown.

## Discussion

*Y. pestis* is a human and animal pathogen responsible for the epidemics of bubonic and pneumonic plagues as recently as the twenty-first century [43]. In the U.S., plague is still endemic in the western states in rodents, the main carrier of the pathogen [44]. Pneumonic plague is highly pathogenic in humans and antibiotics must be administered within the first 24 hr post-infection. Due to the natural emergence of antibiotic resistance in bacteria [45] and the potential for a deliberate engineering of resistance in bacteria, the search for non-antibiotic treatments of infectious diseases has gained more attention in recent years [46]. The targeting of virulence systems of pathogens, by rational drug design against a given target, or by random screening of a library of compounds inhibiting the transport of the virulence system, is a new strategy [37], [47].

The current strategies for targeting *Yersinia pestis* and other type III secretion system-utilizing pathogens rely mostly on phenotypic screens [3], [9], [48], [49], [50], [51], [52]. The strategy uses a library of random compounds or a smaller set of molecules and the readout is a blockage of secretion of a defined substrate into the host. The main drawback is the non-specificity of the library targets which necessitates secondary screens for elimination of general toxicity compounds as opposed to those targeting specifically the virulence system. Identification of the mechanism of action of the inhibitors is not straightforward, either, and it usually requires a separate study to differentiate between prevention of assembly of the injectisome and blocking a specific function of a given protein. In addition, due to the random nature of the library, there is a potential that the targets may be mammalian proteins identified during subsequent screening. In contrast, our approach uses a systems biology approach to identify the potential target and validate it in an animal model. The target is made by recombinant means and the knowledge from structural biology and computational chemistry is used to computationally select for molecules interfering with a specific functional activity of the protein. The molecules are computationally selected for binding to a defined region on the target molecule and verified *in vitro* for blockage of the function and *in vivo* to confirm their ability to block the bacterial secretion process. The strategy is potentially superior to the phenotypic screens as the search is rational and directed against a very specific target. The approach is typically faster, cheaper, more efficient and, as demonstrated in our work, results in better inhibitors with less experimental screening and potentially lower off-target hits.

We hypothesized that therapeutics should target bacterial ATPases involved in the transport process. In *Y. pestis*, the only T3SS ATPase is the YscN protein and therefore, this protein was selected as a therapeutics target for plague. As expected, the target was validated in a mouse model of bubonic plague. The attenuation, over a million-fold (Table 1), of the *ΔyscN* mutant pathogen was not caused by slower growth of the mutant (Fig. 2). Due to the physical limitations of the animal model, higher attenuation levels could not be determined. The results suggest that the potency of future therapeutics directed against YscN may approach levels observed for the currently-used antibiotics.

To help facilitate the development of therapeutics, the optimized catalytic domain of YscN protein was purified from *E. coli* as a fusion with MBP and characterized in detail. The purified protein was catalytically active but also very prone to aggregation. The homologous full-length YscN ATPase from *Y. enterocolitica* was a mixture of different tertiary forms, from monomer to hexamer, but started forming aggregates with a stoichiometry higher than six when examined in the presence of a non-hydrolysable ATP analog [35]. In the enteropathogenic *E. coli*, the homologous full-length EscN ATPase eluted as a mixture of mostly monomeric and 12–14-mer species in the presence of near-saturating substrate concentrations [34]. Obviously, the T3SS ATPases oligomerize easily and the optimized YscN catalytic domain from *Y. pestis* may simply be more prone to aggregation than other proteins.

While some compounds exhibited average to poor values of IC_50_, most of these were effective at blocking of YopE secretion at 100 µM concentration. Explanation of this conundrum may take form of several possibilities. As noted above, we speculate that one possibility is that these compounds hinder hexamer formation of YscN and its consequent mechanical function, rather than inhibit the catalytic turnover of ATP. Unlike the association of ADP to YscN, which helps to regulate oligomerization, molecular modeling of the hexamer suggests that the docked conformational poses of the small molecules may interfere through steric interactions with the correct orientation of adjoining protein chains and their alignment in the multimeric assembly. Specifically, the N-terminal part of the YscN protein was predicted to be essential for the hexamer formation. It is also plausible that the truncated form of YscN used in the inhibition assay is not a true functional mimic of the full chain and its activity in the oligomeric state, thus yielding a weak correlation between the ATPase inhibition and YopE secretion data. A final possibility is that there may be additional binding sites on YscN that can accommodate the small molecules and/or perhaps there are off-target proteins with sufficient sequence similarity with YscN (e.g., type III secretion system ATP synthase) to bind the small molecule inhibitors and disrupt the transport of virulence factors. Nonetheless, even though our goal of *in silico* screening was for enzymatic inhibitors, we discovered a set of molecules that block secretion through perhaps an alternative mode of action. The exact details at atomic-level resolution of how these molecules fully work remain to be determined from X-ray crystallographic studies and further protein-binding assays.

The differences between the *in vivo* and *in vitro* data were investigated in more detail. The most likely explanation, inhibition of bacterial growth, was proven not to be the cause for any of the selected compounds (Fig. 7) except for the compound 7086. An alternative explanation, bioavailability, was investigated indirectly by screening the YopE secretion inhibition at 10 µM concentration of inhibitors (Table 4). While for many of the molecules there was still not a clear correlation between the *in vivo* and *in vitro* data, three of the top ten scoring inhibitors from the activity assay were confirmed in the *in vivo* assay: compounds 7812, 7832 and 7086. Compound 7086 may additionally exert its effect through a slight inhibition of bacterial growth (3 hr time point in Fig. 7). The compounds were also non-toxic (7812 and 7832) or only slightly toxic (7086) to the mammalian cells at ten-fold higher concentrations (Table 5), suggesting that the molecules have a low potential to target human enzymes, ATPases in particular. A full evaluation of the therapeutic potential will require a separate study in the future. However, the low toxicity against human cells shown in the current work is very important for future therapeutics development.

The final test of potential therapeutic applications of the small molecules was performed in the cell culture model of infection. The best overall scoring molecules capable of inhibiting both YopE secretion and ATPases activity (7086, 7812 and 7832) were only moderately effective in preventing the morphological changes in infected HeLa cells (Table 6). However, the best inhibitor in the cell culture model, compound 3624, was relatively potent in the second YopE secretion assays (60% at 10 µM). Since the compound was relatively non-toxic to HeLa cells (20% at 100 µM), the molecule could be optimized in future to increase potency and lower toxicity.

The identified compounds are novel and have not been tested in similar studies [3], [9], [48]. However, a search of chemical databases identified many analogs of the top scoring compounds from different assays performed in the current work (Table S1). Elucidation of the mechanism of action of the top scoring compounds and their full therapeutic potential is beyond the scope of the current work.

Our results show that the strategy to block virulence of human pathogens can be based on small molecules targeting motor proteins of bacterial secretory systems. The proteins are easy to identify by bioinformatics methods and shown to be essential for virulence in many systems. It is possible that the strategy presented in the current work could be used to develop novel therapeutics for many other T3SS-encoding pathogens, including *Salmonella typhimurium*, EPEC/EHEC, *Shigella flexneri*, *Campylobacter jejuni*, *Pseudomonas aeruginosa*, *Burkholderia mallei* and many others.

## Materials and Methods

### Bacterial strains and media

The following reagent was obtained through the NIH Biodefense and Emerging Infections Research Resources Repository, NIAID, NIH: *Yersinia pestis*, Strain KIM Derivative 19 (D19), NR-4681, *Yersinia pestis*, Strain KIM Derivative 2 (D2), NR-4682. For routine growth of the fully virulent pathogen, *Y. pestis* was maintained on sheep blood agar plates or in heart infusion broth (HIB) with 0.2% xylose. For mutant construction, *Y. pestis* was incubated at 28°C on Luria-Bertoni (LB) Lennox plates supplemented with ampicillin at 50 µg/mL or 5% sucrose. For determining colony-forming units (CFU), the bacteria were incubated on sheep blood agar plates (SBAP). The *Y. pestis* KIM19 strain was propagated on sheep blood agar plates and small (3–10 ml) bacterial cultures for YopE secretion controls were made in HIB supplemented with 0.2% xylose.

### Chemicals and reagents

Most of the common chemicals were purchased either from Sigma Aldrich or Fisher Scientific. Protease inhibitors cocktails without EDTA were purchased from Roche Diagnostics. Small molecule compounds identified in the computational screen of ZINC database were purchased either from Life Chemicals or from Enamine as dry powders. Stock solutions of the compounds in dimethylsulfoxide (DMSO) and/or dimethylformamide (DMF) were made in-house. The stocks were stored at −20°C in amber glass vials. Serial dilutions of the stocks with DMSO were made from the thawed stocks when required and stored at −20°C in amber glass vials as well. YopE antibody and the positive control antigen were purchased from Santa Cruz Biotechnology and the mouse anti-goat antibody HRP conjugate was purchased from Thermo Scientific.

### Suicide vector construction

The *yscN* gene fragment, basepairs (bp) 1–300 and 1020–1317, together with about 1 kb extensions on both 3′ and 5′ ends, was amplified by overlap extension PCR [53] using genomic DNA from *Y. pestis* CO92 as a template. The full construct was cloned into the suicide vector pCVD442 [54] and transformed into a pir^+^ strain TransforMax EC100D pir^+^ electrocompetent cells (Epicentre Biotechnologies, Inc.) by electroporation. The *yscN* gene deletion in the construct, bp.301–1019, and inclusion of the overlapping 1 kb fragments on each side of the *yscN* gene, was verified by PCR and DNA agarose gel electrophoresis.

### Gene deletion in *Y. pestis CO92*

To inactivate the *yscN* gene from the pCD1 plasmid of CO92 strain of *Y. pestis*, the plasmid containing the mutant *yscN* (Δ*yscN* long) was introduced by electroporation [55] using a (BioRad Laboratories) MicroPulser Electroporator with 0.1 cm cuvettes. Cointegrates were selected on LB agar plates in the presence of 50 µg/mL of ampicillin. The cointegrate strains were grown overnight in HIB and plated on LB agar plates containing sucrose to select for allelic exchange recombinants, as pCVD422 contains the *sacB* gene [56]. Those colonies which grew in the presence of sucrose were screened by PCR to demonstrate the deletion of the *yscN* gene. To confirm that the virulence plasmid was still present in the Δ*yscN* CO92 strain and not lost during *in vitro* growth, a primer pair set directed against the V-antigen was utilized for PCR amplification to demonstrate its presence (data not shown). Likewise, the Δ*yscN* mutant strain was screened on Congo Red [57] agar plates to confirm the *pgm* locus was intact.

#### *Yersinia pestis Δ*yscN growth curve determination

To determine the growth profile of the Δ*yscN* mutant, overnight cultures of the wt and mutant strains in HIB supplemented with 0.2% xylose were inoculated into fresh medium at the cell density corresponding to OD_620_ of approximately 0.05 and grown at 28°C at 150 rpm. The growth of bacteria was then monitored by measuring OD_620_ for up to 20 hrs.

The *Y. pestis* wild-type and Δ*yscN* strains were prepared for mouse inoculations as previously described [58], except that the bacteria were suspended in 10 mM potassium phosphate buffer saline (KPBS) solution rather than HIB. Groups of 10 naïve female Swiss-Webster mice were inoculated via the subcutaneous route with 0.2 mL aliquots of the Δ*yscN Y. pestis* mutant at the following doses: 0, 10^2^, 10^3^, 10^4^, 10^5^, 10^6^ and 10^7^ CFU. The intermediate data for survival after 21 days post-inoculation are shown in Table 1 for the purpose of the current work. The data are a part of a separate experiment which was performed over 65 days in total and the results will be published in a separate manuscript. After 65 days, the survivors were humanely euthanized.

For control experiments, the non-inoculated animals were challenged with 180 CFU (approximately 90 LD_50_) of the wild-type *Y. pestis* CO92 strain via the s. c. route. The animals were observed for 21 days after which survivors were humanely euthanized.

### Statistics

To determine differences between the immunized groups and control group, the following determinations were made. Survival rates were compared by Fisher exact tests with stepdown Bonferroni adjustments. The above analyses were conducted using SAS Version 8.2 (SAS Institute Inc., SAS OnlineDoc, Version 8, Cary, NC 2000).

### YscN gene cloning

The synthetic gene, corresponding to the amino acid (a.a.) 95–419 of the *Y. pestis* YscN protein (Figure S1) was optimized for *E. coli* expression *in silico* and assembled using standard synthetic chemistry methods (GenScript). The gene was cloned into pMAL-c4E expression vector (New England Biolabs) between EcoRI and HindIII sites and the final construct was verified by DNA sequencing. The final construct coded for a cleavable (enterokinase) N-terminal fusion with a maltose binding protein (MBP) and a non-cleavable C-terminal 10×His affinity tag. The separation of tags was designed to minimize protein degradation during purification. For protein expression, the clones were transformed into BL21 (DE3) expression strain (EMD), verified by SDS-PAGE to express the desired protein, and stored as glycerol stocks at −80°C.

The bsaS gene (*B. mallei* ATCC 233344) coding for BsaS protein fragment corresponding to amino acids (a.a.) 18–433 (Figure S2) was optimized for *E. coli* expression *in silico* and assembled (GeneScript) as described for the *Y. pestis yscN* gene. The final construct was cloned in pMAL-p5E vector (NEBiolabs) between KpnI and BamHI sites. The corresponding ORF of *bsaS* gene had all Cys residues replaced by Ser. The plasmid construct was transformed into NEB Express (NEBiolabs) *E. coli* strain for protein expression.

### Protein expression and purification

To express the YscN protein fragment, stock of the verified clone was streaked onto a fresh LB+ampicillin (50 µg/mL) agar plate and grown overnight at 37°C. A single colony was selected to inoculate 3 mL of LB+ampicillin (50 µg/mL) medium. The cells were grown overnight at 37°C in an incubator with shaking at 200 rpm. The next day, the cell culture was diluted 1∶10 into a fresh medium and incubated at 37°C with shaking for 2 hr. The culture propagation was continued until the final dilution, typically 2–4 L of Overnight Express Instant TB medium (EMD) supplemented with antibiotic (50 µg/mL ampicillin), was reached. The culture was incubated for an additional time until the cell density reached OD_600_ = 0.4–0.6. At that time, the culture was chilled to 22°C in the shaker and a solution of isopropyl-β-D-thiogalactopyranoside (IPTG) was added to a final concentration of 0.3 mM. The culture resumed shaking for an additional 16–20 hr. Cells were harvested by centrifugation was and stored at −20°C (short term) or −80°C (long term) before extraction of proteins.

To purify proteins, the cell paste was thawed at room temperature (25°C) for about 30–60 min, resuspended in chilled 200–400 mL of protein binding buffer (10 mM Tris, pH = 8.0, 150 mM NaCl, 10% glycerol, 40 mM imidazole) supplemented with Complete EDTA-free (Roche Diagnostics) protease inhibitor cocktail. The bacterial paste was homogenized using the M-110-P 20 Microfluidizer (Microfluidics), the debris removed by centrifugation, the supernatant filtered through a sterile 0.45 µm filter and purified on AKTAExpress Single (GE Healthcare) automated protein purification chromatography system using a three-step protocol: Ni-agarose (HisTrap Crude FF 5 mL), gel filtration (HiLoad Superdex 200 26/60) and amylose resin (MBP Trap HP 5 ml) according to the standard methods. The protein fractions were buffer exchanged into 10 mM Tris, pH 7.6, 150 mM NaCl, 10% glycerol and concentrated to 0.6–0.9 mg/ml using the Amicon Ultra-15 concentrators (Millipore). Protein concentration was determined with an *RC DC* Protein Assay kit II (Bio-Rad Laboratories) with bovine serum albumin (BSA) as a standard. The concentrated protein was distributed into aliquots and stored at −20°C for experiments. Typically, 1–2 mg of purified YscN protein was obtained from 1 L of bacterial cell culture.

The procedures for purification of BsaS protein were identical to the procedures described for the YscN protein with one exception: the bacterial culture was scaled up to 10 L to compensate for the low expression levels of the recombinant MBP-BsaS protein fusion.

### Comparative protein modeling of YscN

Because of the lack of an experimental structure of YscN ATPase from either X-ray crystallographic or NMR determination, a comparative protein model was developed at atomic-level resolution. Protein structure prediction of YscN was carried out by using the protein homology/analogy recognition engine (Phyre) server [59]. Twenty hits were culled from profile-profile threading of the YscN sequence through a database of known protein folds and the top-ranked match yielded the protein PDB ID: 2dpy, which is an X-ray crystal structure of a flagellum-specific ATP synthase in the ADP-bound form. From the Phyre alignment, the computed pairwise sequence identity between YscN and 2dpy was 45%, thus enabling an accurate three-dimensional comparative model to be constructed for YscN. Hydrogen atoms were added to the comparative model by the CHARMM package [60].

### Computational screening of ZINC database

The ZINC chemical database (version 8) [36] was searched by virtual screening and the database contained approximately 5 million commercially available compounds with predicted drug-like chemical properties. Small-molecule docking of the Zinc library to the YscN protein model was achieved by using the DOVIS large-scale virtual screening pipeline [61]. The docking engine implemented in DOVIS is AutoDock 4.0 [62] and for the application presented here, we modeled the YscN protein as a rigid-body molecule while the ligands were allowed to be flexible around torsional angles. The energy grid center was defined as the geometrical center of the YscN ADP-binding site (2dpy reference frame) at (x = 8.0, y = 4.4, z = 41.0) with a volume of 30×20×20 Å^3^ at a grid-resolution spacing of 0.375 Å. AutoDock parameters were set as follows: 10 genetic algorithm runs were executed, each with population size of 150, one million energy evaluations, and a maximum of 27,000 generations per genetic algorithm run. Three clusters were saved per ligand with a root-mean-square-deviation of at least 1.5 Å. The default AutoDock 4.0 scoring function was used to select the top three clusters of conformational poses for each ligand and to down-select the top 20,000 compounds from the sampled 5 million.

For each of the top 20,000 compounds ranked by AutoDock 4.0 score, the top three docked complexes were minimized with 200 steps of the adopted basis Newton-Raphson method using CHARMM [60] with the MMFF force field [63] and a distance-dependent dielectric solvent term (ε = 4). OpenBabel software was used to interconvert between SDF, MOL2, and PDB format as required. The minimized complexes were re-scored with LigScore2 (Accelrys, Inc.) [64]. The top 50 compounds ranked by LigScore2 that were available from the commercial vendors Enamine and Life Chemicals were selected and purchased.

### Enzyme activity assays

#### Phosphate release assay

The EnzCheck Phosphatase Assay kit (Invitrogen, CA) was used in the initial screening for YscN ATPase activity. The determination of released phosphate was performed with the EnzCheck Phosphatase Assay kit (Invitrogen, CA) according to manufacturer guidelines. Data analysis and graphing were performed with KaleidaGraph (Synergy Software, PA) software.

#### ADP release assay

The Adapta Universal Kinase kit (Invitrogen, CA) was used to screen for inhibition of YscN and BsaS ATPase activities by the small-molecule inhibitors derived from computational screen. Due to the relatively low activity of the recombinant enzymes, the assay was modified for low concentrations of ATP in the reaction mixture as described below.

#### ADP release assay calibration

To maximize the ability of the assay to detect ATPase inhibitors at 10 µM ATP working concentration, the amount of tracer used in the assay was equal to the amount recommended by the manufacturer for 1 µM ATP concentration. The samples were analyzed using the Synergy 4 (Biotek, Inc.) microplate plate reader according to the manufacturer's instructions. The optimal concentration of the protein selected for inhibitor screening corresponded to the amount required to elicit an approximately 80% change (EC_80_) in the assay signal. The EC_80_ determination was performed for each lot of the recombinant enzyme.

#### Preliminary molecule screening for ATPase activity inhibition

The potential inhibitors were resuspended in a mixture of DMSO and DMF to a standard concentration of 50 mg/mL. Due to the solubility problems of the small molecules, only 37 out of the total of 50 compounds could be used for making stock solutions at 50 mg/ml. The insoluble compounds were not used for further testing. For screening, the inhibitors were diluted to 5 mg/mL in DMSO and the ADP release was analyzed using the Adapta Universal Kinase kit (Invitrogen, CA) as described.

#### Determination of IC_50_ values

Compounds demonstrating near-complete inhibition of ADP release at 100 µM concentration were serially diluted 1∶10 in DMSO and assayed for inhibition at 100, 10, 1, and 0.1 µM final concentrations. Since all of the compounds were non-inhibitory below 1 µM final concentration, each inhibitor was tested at 0.7–100 µM final concentration range. The data were imported into KaleidaGraph (Synergy Software, PA) software for analysis. The *IC_50_* values were determined from the sigmoid-fitting model supplied with the software.

#### Screening of small molecules for inhibition of YopE secretion in a bacterial cell culture

The secretion of YopE protein was performed in a method similar to one previously described [65]. An overnight culture of *Y. pestis* KIM19 was grown from a single colony in 25 mL of HIB medium supplemented with 2.5 mM CaCl_2_ and 0.2% xylose at 26°C. The culture was then diluted to an OD_600_ of approximately 0.2 in fresh medium and split into 5 mL samples. Various YscN inhibitors were added to the separate culture tubes, each at concentration of 100 µM. A negative control was DMSO without inhibitor. The cultures were then allowed to grow at 26°C for 1 hr and the temperature was shifted to 37°C for an additional hour. After that time, 500 µl samples were taken to provide a baseline measurement of YopE secretion and EGTA was added to a concentration of 10 mM in all but the negative control. The cultures were returned to the incubator for additional 1 hr incubation at 37°C. At the end of the incubation, another set of 500 µL samples were removed from the cultures, centrifuged immediately at 16,000 rpm for 5 min. and the supernatants were passed through 0.22 µM filters. The detection of secreted YopE was performed by ELISA.

#### Determination of secreted YopE in cleared bacterial culture supernatants by ELISA

Samples from the YopE secretion assay were analyzed either undiluted or at a 1∶10 dilution in water. The primary antibody was goat anti-YopE (1∶500 dilution) and the secondary was mouse anti-goat HRP conjugate (1∶3000 dilution). The visualization was performed with the ABTS peroxidase solution by reading absorbance at 405 nm on the Synergy 4 (BioTek, Inc.) microplate reader. Data manipulation and plotting were performed with KaleidaGraph software (Synergy Software).

### Inhibition of mammalian cell culture toxicity by selected small molecules

#### HeLa cell preparation

Two days prior to infection with attenuated *Y. pestis* strain, 200 µL of 3.5×10∧4 cells/mL of HeLa cells in DMEM medium containing 10% FBS were placed into a 96 well plate. The plates were incubated for 2 days at 37°C with 5% CO2.

#### Bacterial cell preparation

Two to three days prior to the infection of mammalian cells, a blood agar plate was streaked from a frozen stock of *Y.pestis* KIM19, containing the pCD1 plasmid, and KIM2, lacking the pCD1 plasmid, and incubated at 26°C. The day before infection, 3 mL of HIB broth was inoculated with a single colony from the blood agar plate and incubated at 26°C while shaking at 300 rpm. The following morning, a 5 µL aliquot of the *Y. pestis* culture was used to inoculate 500 µL of DMEM medium supplemented with 3% FBS and the inhibitor at a concentration of 50 or 10 µM dissolved in DMSO. The final concentration of DMSO in the culture was 1%. These cultures were incubated for 1 hour at 26°C while shaking at 200 rpm.

#### Infection of mammalian cells

After a one hour of incubation of HeLa cells with *Y. pestis*, the plates containing the HeLa cells were removed from the incubator and the media was removed by aspiration. A 150 µL aliquot of the various cultures containing the inhibitors or DMSO control were placed into duplicate wells and the plates were incubated for 3 hours at 37°C with 5% CO2. Following the incubation, the plates were removed from the incubator and analyzed by optical microscopy. The number of rounded and squamous cells was counted manually and the percent of squamous cells was compared to the controls.

### Ethics statement

Research was conducted in compliance with the Animal Welfare Act, and other federal statutes and regulations relating to animals and experiments involving animals, and adheres to principles stated in the Guide for the Care and Use of Laboratory Animals, National Research Council, 1996. The facility where this research was conducted is fully accredited by the Association for Assessment and Accreditation of Laboratory Animal Care International. The research protocol was approved by the Laboratory Animal Care and Use Committee (approved animal protocol number: AP-08-010). Opinions, interpretations, conclusions, and recommendations are those of the authors and are not necessarily endorsed by the United States Army.

## Supporting Information

Figure S1 **Sequence of optimized gene coding for the YscN protein fragment a.a. 95–419.** Restriction sites EcoRI and HindIII are marked in red.
(TIF)

Figure S2 **Sequence of optimized gene for the BsaS protein fragment a.a. 18–433.** KpnI and BamHI restriction sites are marked in red.
(TIF)

Table S1 **Putative targets for selected inhibitors of YopE secretion.**
(DOCX)

Appendix S1 **List of 2D structures and the corresponding in-house ID numbers.** Each small compound has an associated in-house ID number listed under the 2D structure.
(PPTX)
