# Supplementary figures and images for: Identification of Small-Molecule Inhibitors of Yersinia pestis Type III Secretion System YscN ATPase
Source: PLoS One. 2011 May 18;6(5):e19716. doi: 10.1371/journal.pone.0019716 (PMC3097197; doi:10.1371/journal.pone.0019716)

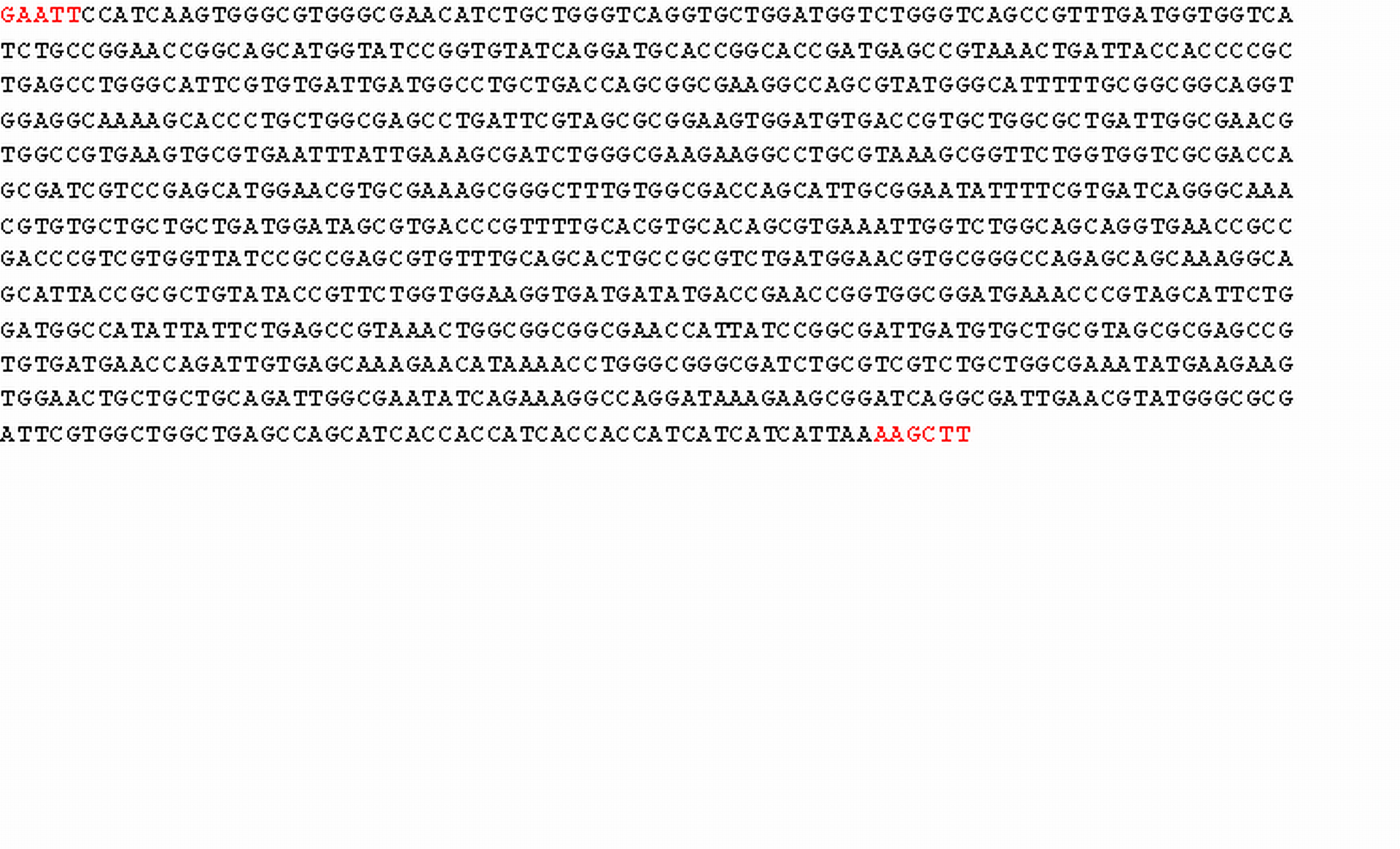

Supplement: Figure S1 — Sequence of optimized gene coding for the YscN protein fragment a.a. 95–419. Restriction sites EcoRI and HindIII are marked in red. (TIF) [file pone.0019716.s001.tif]

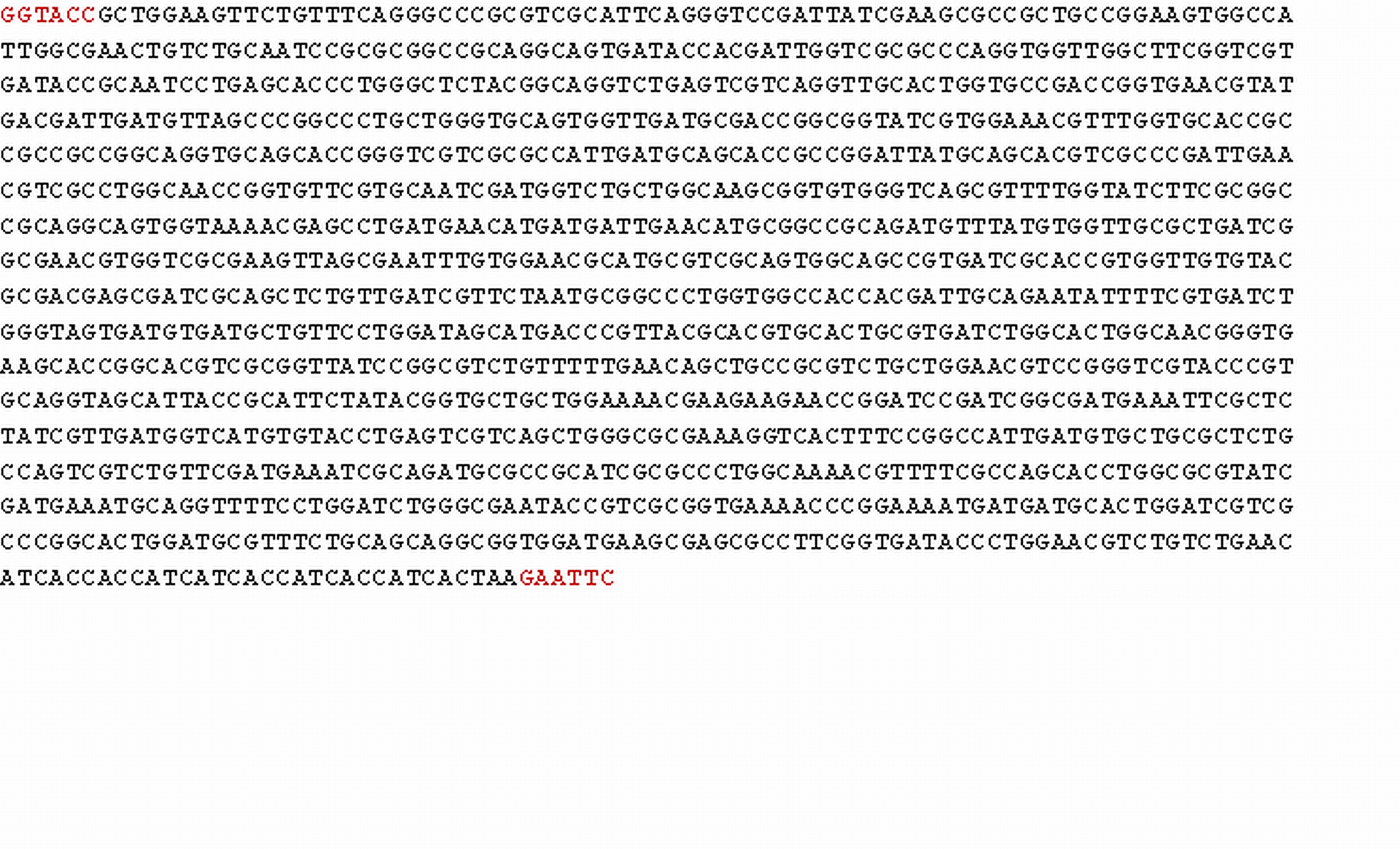

Supplement: Figure S2 — Sequence of optimized gene for the BsaS protein fragment a.a. 18–433. KpnI and BamHI restriction sites are marked in red. (TIF) [file pone.0019716.s002.tif]

## Slide 1
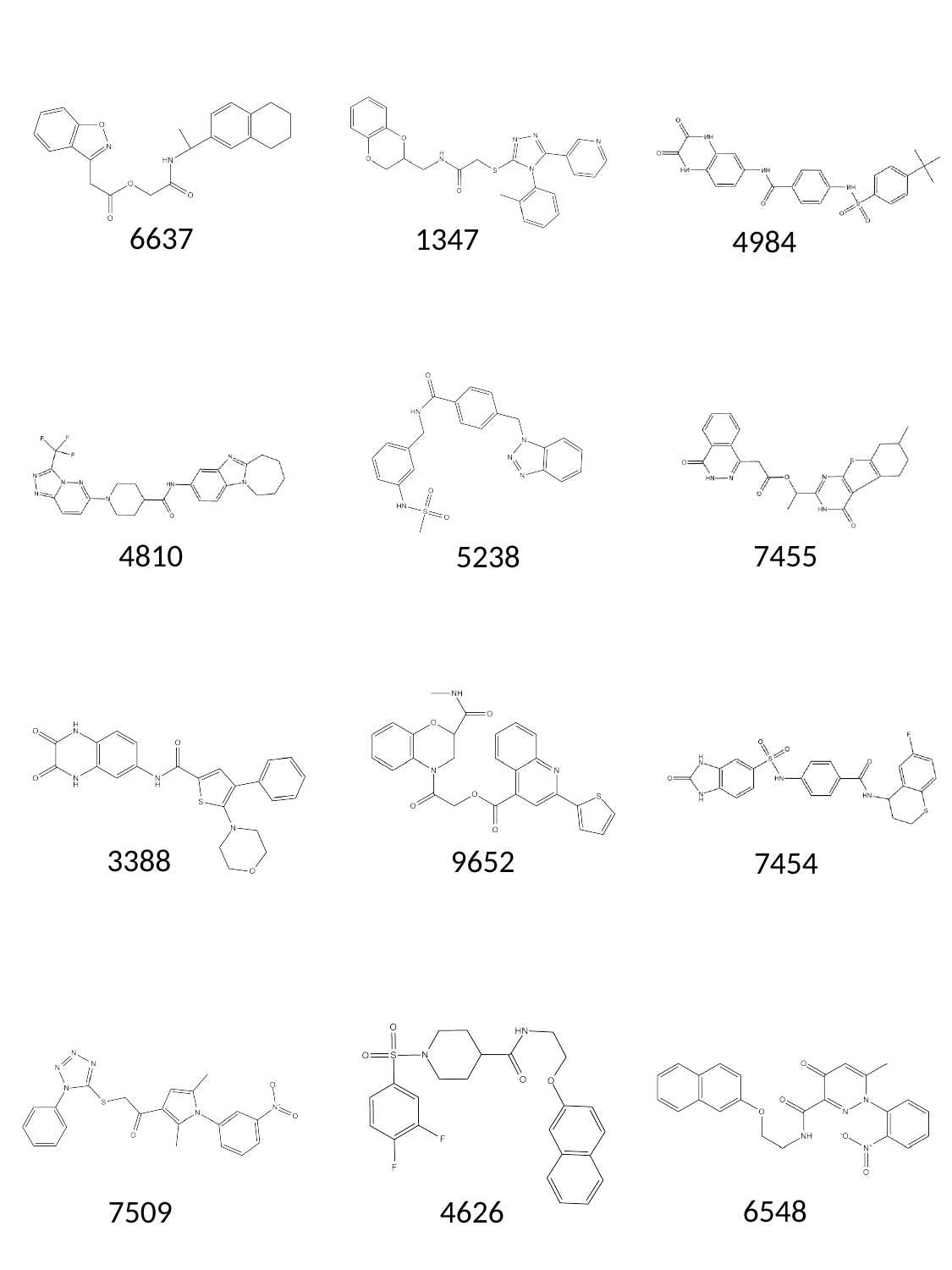

| | | |
| --- | --- | --- |
| | | |
| | | |
| | | |
6637
1347
4984
4810
7455
5238
3388
9652
7454
6548
7509
4626

## Slide 2
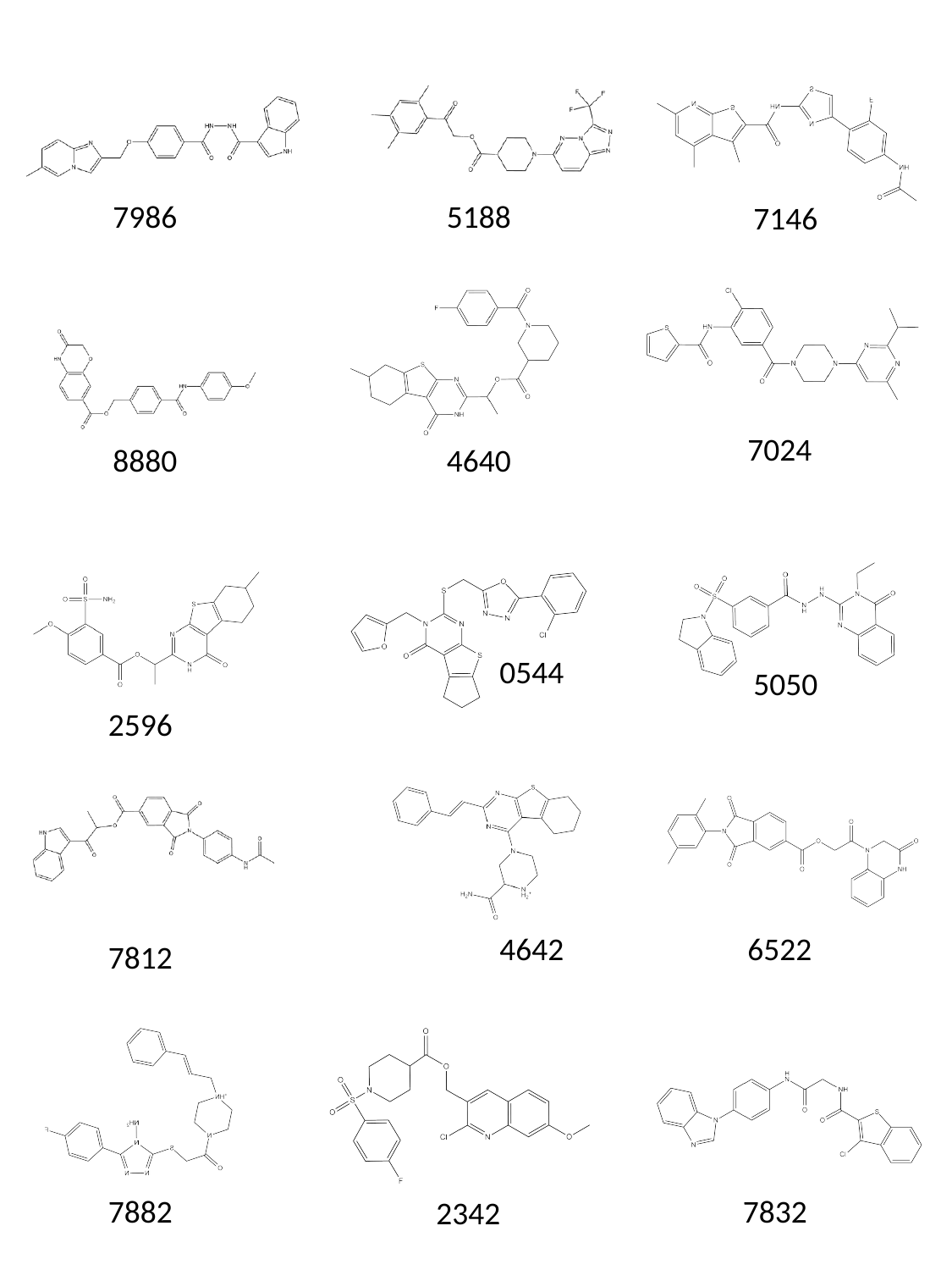

| | | |
| --- | --- | --- |
| | | |
| | | |
| | | |
7986
5188
7146
7024
8880
4640
0544
5050
2596
4642
6522
7812
7882
7832
2342

## Slide 3
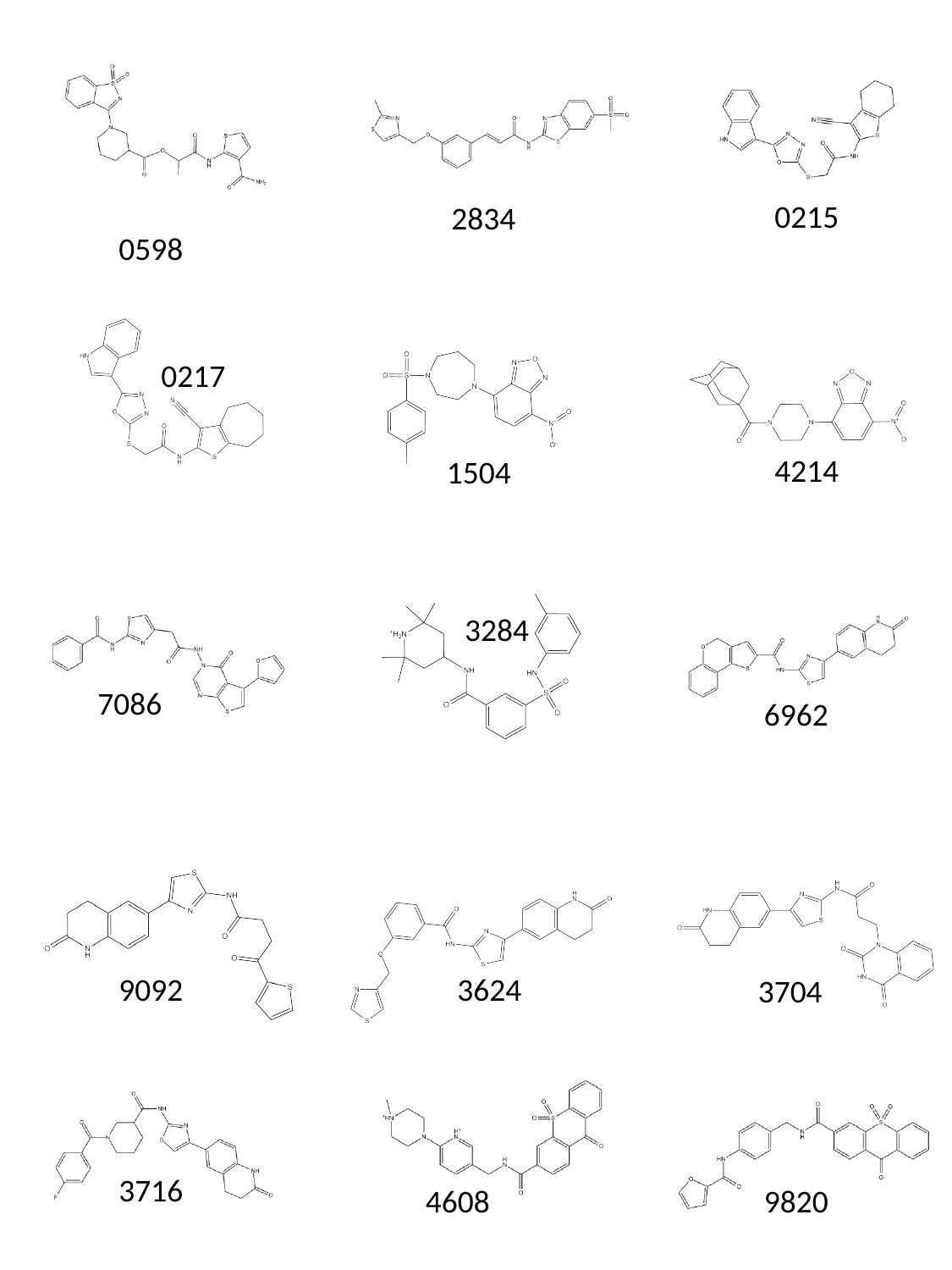

| | | |
| --- | --- | --- |
| | | |
| | | |
| | | |
0215
2834
0598
0217
4214
1504
3284
7086
6962
9092
3624
3704
3716
4608
9820

## Slide 4
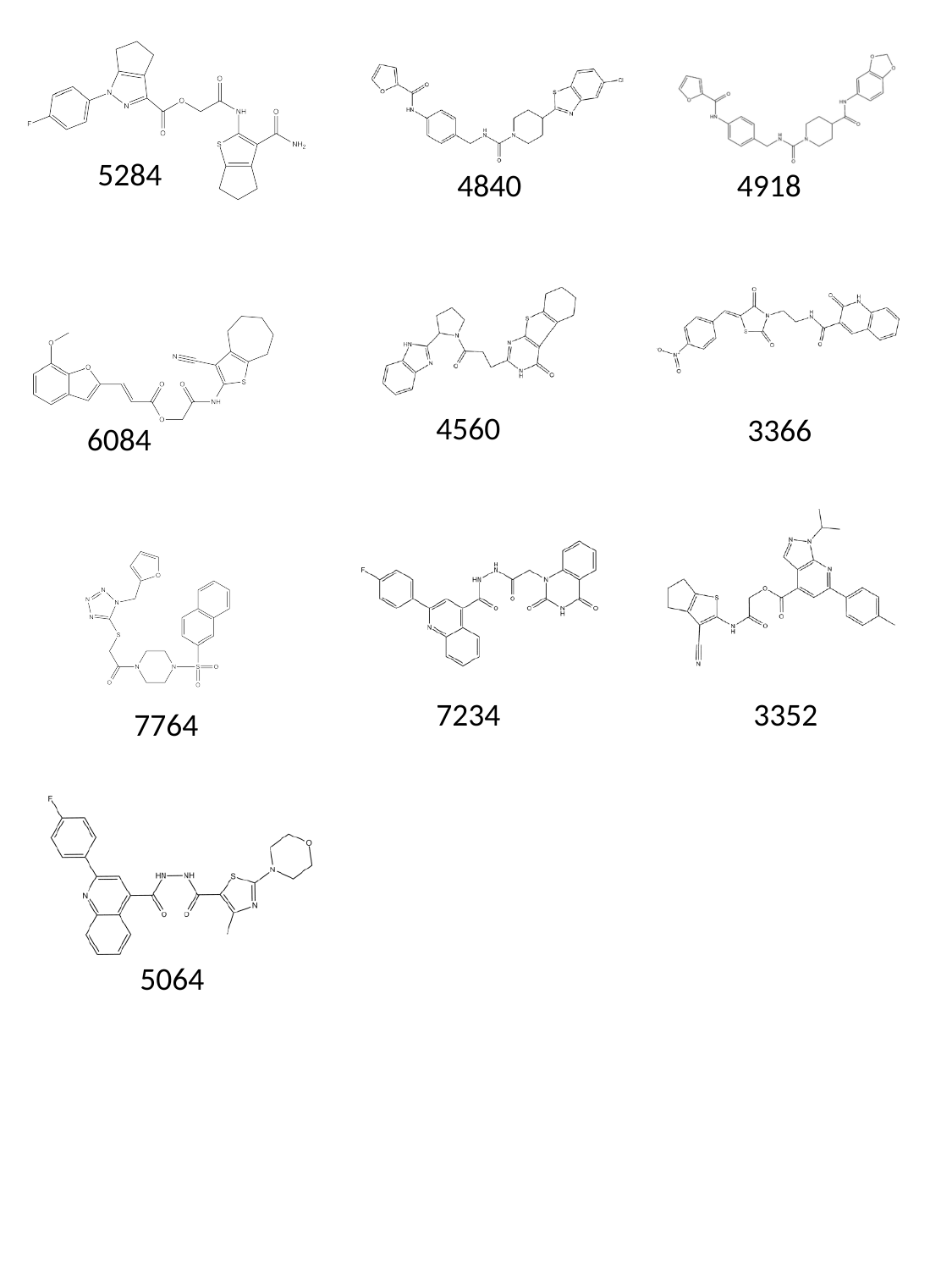

5284
4840
4918
4560
3366
6084
7234
3352
7764
5064

Supplement: Appendix S1 — List of 2D structures and the corresponding in-house ID numbers. Each small compound has an associated in-house ID number listed under the 2D structure. (PPTX) [file pone.0019716.s004.pptx]
