# Supplementary material for: Identification of Small-Molecule Inhibitors of Yersinia pestis Type III Secretion System YscN ATPase
Source: PLoS One. 2011 May 18;6(5):e19716. doi: 10.1371/journal.pone.0019716 (PMC3097197; doi:10.1371/journal.pone.0019716)
Supplement: Table S1 — Putative targets for selected inhibitors of YopE secretion. (DOCX) [file pone.0019716.s003.docx]

**Table S1**. *Putative targets for selected inhibitors of YopE secretion*

| Compound | Putative Target |  |
| --- | --- | --- |
| *In-house ID* | *Similarity ensemble approach (SEA) prediction* |  |
|  |  |  |
| 6962 | CCK A antagonist |  |
| 7146 | CCK A antagonist |  |
| 7024 | Phospholipase C inhibitor |  |
| 4626 | Endothelin formation inhibitor |  |
| 7509 | Elastase inhibitor |  |
| 3716  3624 | Antithrombocytopenic  Antithrombocytopenic |  |
| 3284 | HT1F antagonist |  |
| 5064 | Calpain inhibitor |  |
| 6084  2834  7812  7832  7086 | Agent for nefritis  Mediator release inhibitor  HTF1 agonist  Beta-lactam enhancer  N/D |  |
|  |  |  |

The SEA predictions were based on the search results of MDL Drug Data Report 2006.1 database of known targets [59]. Reported targets are the top-scoring hits. N/D – not determined.
